# Volume Deformation Control of Concrete for Hydraulic Structures Using Polyurethane-Modified Polycarboxylate Superplasticizer: A Review

**DOI:** 10.3390/ma19081648

**Published:** 2026-04-20

**Authors:** Benkun Lu, Jie Chen, Shuncheng Xiang, Zhe Peng, Changyu Liu, Yafeng Ouyang, Yuelin Li, Jing Zhang

**Affiliations:** 1 School of Hydraulic and Ocean Engineering, Changsha University of Science & Technology, Changsha 410114, China; 19330787631@163.com (B.L.); 13054166813@163.com (S.X.); 13187274362@163.com (Y.O.); 19898822758@163.com (Y.L.); 17338233987@163.com (J.Z.); 2 Hunan Provincial Department of Transportation, Changsha 410004, China; 3 Key Laboratory of Water-Sediment Sciences and Water Disaster Prevention of Hunan Province, Changsha 410114, China; 4 Hunan Provincial Water Transport Construction & Investment Group Co., Ltd., Changsha 410029, China; 19892933769@163.com (Z.P.); 15279937190@163.com (C.L.)

**Keywords:** polycarboxylate, concrete, volume deformation, surface tension

## Abstract

As a widely used building material, the performance of concrete has a far-reaching impact on the quality and durability of hydraulic engineering. Polycarboxylate superplasticizer (PCE) plays an increasingly important role in concrete engineering because of its unique high-efficiency water-reducing performance and the improvement effect on concrete performance. In this paper, the application and influence of polycarboxylate in concrete, including its chemical structure, action mechanism and application effect, are reviewed. It is found that polycarboxylate can greatly reduce the shrinkage of concrete and control its volume deformation. The objective of this review is to elucidate the mechanisms by which polyurethane-modified polycarboxylate (MPCE) reduces autogenous and drying shrinkage in concrete and to demonstrate its advantages over conventional PCE. On this basis, we focus on the core research object of MPCE and discuss in depth its effect on reducing the surface tension of concrete pore solution and the intrinsic mechanism of regulating volume deformation. The research clarifies the superior performance of MPCE over ordinary PCE in inhibiting autogenous shrinkage and drying shrinkage in concrete, which provides a targeted scientific basis for the practical application of MPCE in concrete volume deformation control.

## 1. Introduction

Hydraulic concrete is mainly used in hydraulic engineering, marine engineering and other environments. Its definition depends on the combination of concrete properties, encompassing heat release, and volume change (such as thermal deformation strain, shrinkage strain, and creep strain), and the development of physical properties (particularly Young modulus, compressive strength, and tensile strength) of concrete [1,2,3]. Experimental studies often employ relatively thin high-performance concrete elements to simulate realistic cross-sections, leveraging their rapid stiffness development and high heat release characteristics. The complex and harsh service environment of concrete imposes stringent requirements on its durability and crack resistance. Polycarboxylate superplasticizers significantly enhance the density and uniformity of concrete while improving its flowability without increasing the water demand, thereby mitigating volume deformation and strength loss under external exposure [4]. For example, it has been reported that the addition of polycarboxylate superplasticizer can reduce the water demand of concrete by approximately 25–30% while maintaining the same workability, depending on the dosage and cement type [5]. By improving the initial strength and impermeability of concrete, a concrete structure with good structural performance can be formed in a short period of time, improving construction efficiency and reducing maintenance costs.

While Portland cement is inherently hydraulic, the term “hydraulic concrete” is used in the title to specify the application context. In the following text, we generally use “concrete” for brevity, with the understanding that it refers to concrete intended for hydraulic engineering applications.

Polycarboxylate ether (PCE) superplasticizers are comb-shaped copolymers. Their molecular structure typically consists of a main backbone formed via free-radical polymerization of (meth)acrylic acid or related monomers bearing carboxylate functional groups, which enable adsorption onto the surface of cement particles. Grafted onto this backbone are polyethylene glycol (PEG) or other polyether side chains, which extend into the aqueous phase and generate steric hindrance [6]. The molecular structure of polycarboxylate enables effective adsorption onto cement particle surfaces, increasing inter-particle repulsion and thereby significantly improving concrete flowability without raising water consumption [7]. Polycarboxylates have a significant impact on concrete, and their main function is to improve the flowability of concrete, change the microstructure of concrete, and thus lead to better workability and pumpability [8]. This characteristic is particularly important in concrete construction, as it ensures a uniform distribution of concrete during the pouring process, reduces the possibility of segregation and layering, and reduces labor intensity and construction costs. In addition, polycarboxylates have a significant improvement effect on the compressive and flexural strength of concrete [9]. The improvement of concrete’s microstructure by superplasticizer, resulting in reduced porosity, increased density, and enhanced strength and durability, is attributed to the use of these agents. Polycarboxylate also has a positive impact on the chemical stability and environmental friendliness of concrete. As polycarboxylate superplasticizer can effectively reduce the amount of cement used, this means that energy consumption and carbon emissions can be reduced in the production process of concrete, thereby improving the sustainability of concrete.

Given the characteristics of concrete, exploring methods to regulate its volume deformation is particularly important. In this regard, the modification and design of water-reducing agents stand out as crucial approaches. However, ordinary polycarboxylate ether (PCE) still has obvious limitations in the volume deformation control of concrete: its effect on reducing the surface tension of pore solution is limited, the compatibility with supplementary cementitious materials (SCMs) is poor, and it is difficult to simultaneously inhibit the autogenous shrinkage and drying shrinkage of concrete [10,11,12,13,14,15]. Therefore, in order to achieve a wider and more effective application of PCE in concrete, the molecular modification of PCE is an inevitable research direction. Polyurethane-modified polycarboxylate superplasticizer (MPCE) is a targeted modified product for the volume deformation characteristics of concrete. The introduction of polyurethane side chains optimizes the molecular structure of PCE, which can significantly reduce the surface tension of concrete pore solution, improve the adsorption capacity on cement particles, and enhance the compatibility with SCMs. Over the past two decades, extensive research has been devoted to understanding the volume deformation behavior of concrete and the role of chemical admixtures. Several review articles have summarized the mechanisms of autogenous shrinkage and drying shrinkage [16,17,18,19,20,21], the working principles of polycarboxylate superplasticizers [6,7,22], and the development of shrinkage-reducing admixtures (SRAs) [23,24,25]. However, most existing reviews focus either on concrete shrinkage mechanisms or on conventional PCE chemistry, with limited integration of the two. Moreover, the emerging class of modified polycarboxylates that combine water-reducing and shrinkage-reducing functions has not been systematically reviewed. Specifically, polyurethane-modified polycarboxylate (MPCE)—which addresses the limitations of ordinary PCE by introducing polyurethane side chains—has only recently been reported [26], and its mechanisms for controlling volume deformation have not been comprehensively discussed in the literature. Therefore, a critical gap exists in the current knowledge: a systematic evaluation of MPCE’s performance, its superiority over conventional PCE, and its potential for hydraulic concrete applications is urgently needed.

The most important originalities and contributions of this review are as follows: (i) systematic comparison of MPCE with ordinary PCE and SR-PCE; (ii) elucidation of the dual mechanism of MPCE (surface tension reduction + C-S-H gel protection); (iii) quantitative analysis of MPCE’s compatibility with SCMs (fly ash, slag, metakaolin); (iv) practical recommendations for hydraulic engineering.

Based on this, this paper first systematically analyzes the main causes and core mechanisms of volume deformation of concrete, then describes the molecular design of ordinary PCE and the targeted modification strategy of MPCE, and finally, through experimental research, clarifies the regulation effect and action mechanism of MPCE on the volume deformation of concrete, so as to provide theoretical and experimental support for engineering applications of MPCE.

This paper combines a literature review with experimental validation: it first systematically reviews the mechanisms of concrete volume deformation and the molecular design strategies of PCE, then experimentally verifies the regulation effect of MPCE on volume deformation and provides comparative analysis with other studies.

## 2. Causes of Volume Deformation in Concrete and Theoretical Basis for MPCE Regulation

In ordinary cement concrete, volume deformation occurs due to autogenous shrinkage, drying shrinkage, and thermal contraction caused by a temperature drop. Many experts and scholars have studied the mechanism of volume deformation [16,17,18,19]. In concrete, autogenous shrinkage and drying shrinkage have a more significant impact on volume deformation, and their boundaries are also relatively clear. However, thermal contraction deformation is difficult to define due to the complex interplay between temperature changes within the concrete and the surrounding environmental conditions. Therefore, this article only discusses autogenous shrinkage and drying shrinkage and takes the core influencing factors of the two shrinkage types as the theoretical basis for MPCE regulation, clarifying the targeted action points of MPCE in volume deformation control. Subsequently, the influence of aggressive environments (primarily sulfates and chlorides) on the structural and strength parameters of concrete containing PCE is also discussed.

### 2.1. Autogenous Shrinkage

**Figure 1:**
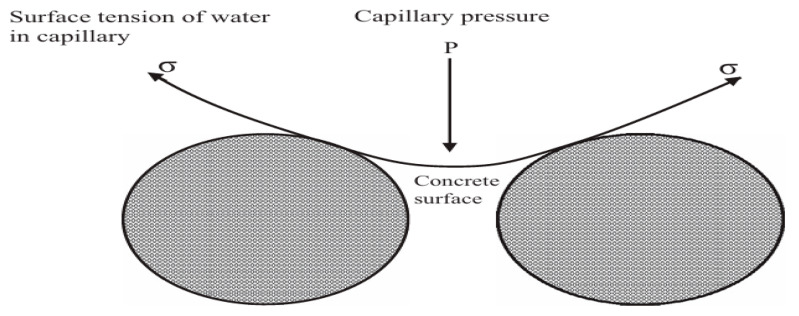
Schematic diagram of capillary water tension.

Autogenous shrinkage refers to the volume reduction of concrete caused by self-desiccation during cement hydration, particularly in mixtures with a low water-to-cement ratio (typically ≤ 0.42). As hydration progresses, internal free water is consumed, leading to a decrease in relative humidity within the capillary pores. The resulting capillary tension generates compressive stress on the pore walls, which manifests as macroscopic volume shrinkage [20]. Wu et al. [21] propose that as cement hydration progresses, free water in the cement paste is gradually reduced, and the internal relative humidity is lowered. This leads to the formation of numerous pores in the hardened cement paste, along with a decrease in pore water saturation. When capillary pores transition from a saturated to an unsaturated state, internal pressure is exerted on the menisci within the pores. To maintain equilibrium of the menisci, capillary tension is continuously increased, thereby inducing autogenous shrinkage. The mechanism of autogenous shrinkage is illustrated in Figure 1.

Due to thermal expansion and contraction, at the beginning of hydration, as the temperature increases, the core temperature of concrete is higher than the outer surface, resulting in relatively higher expansion. The concrete surface cracks due to tensile stress [1]. This occurs because, under external constraints, the temperature rise in the concrete core induces compressive stress, while subsequent cooling generates tensile stress. During the heating phase, the core expands more than the surface, but due to external constraints, this expansion is restricted, leading to the development of compressive stress in the core. After temperature equilibrium, the compressive stress is converted into tensile stress, and at this point, the tensile stress exceeds the initial compressive stress. This phenomenon mainly arises from the varying Young moduli of concrete during the heating and cooling processes. As a result of the high stress relaxation of concrete and low Young modulus during the heat release stage, the initial compressive stress is very low. As the temperature decreases, there is a concurrent increase in the degree of hydration of concrete, which results in a rise in Young modulus. This increase in temperature-induced hydration also correlates with a reduction in stress relaxation, a trend that is inversely proportional to the hydration degree [27].

In conclusion, when the temperature of fresh concrete exceeds the equilibrium temperature of the environment during the cooling process, the resulting temperature difference during the expansion process is smaller compared to the temperature difference experienced during the cooling process. Moreover, the heating and subsequent cooling of concrete induces thermal strain. The coefficient of thermal expansion (CTE) quantifies the strain induced per unit change in temperature. During the early stages of cement hydration, particularly around the time of solidification, the CTE is relatively low. However, as hydration proceeds and self-desiccation gradually develops, the CTE tends to increase.

**Figure 2:**
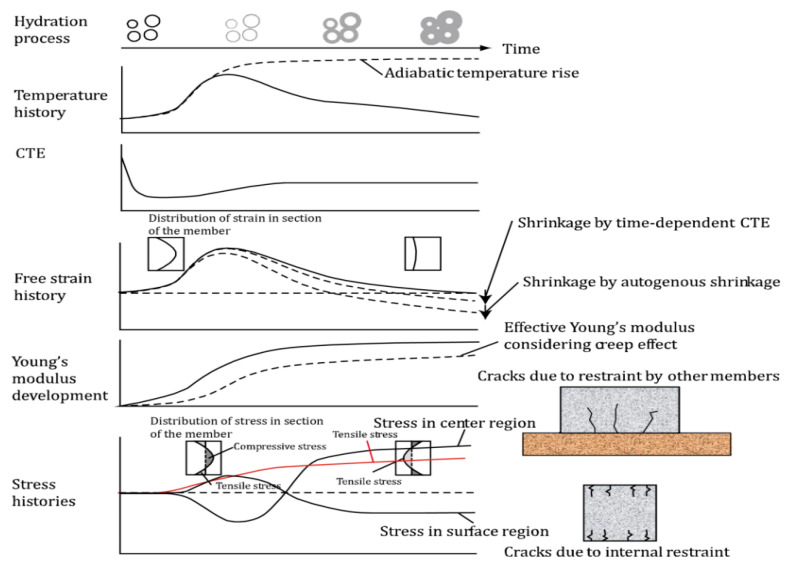
Schematic figure for cracking of concrete.

The CTE of cement concrete is notably lower than that of cement paste and mortar. This difference is attributed to the lower CTE of rigid aggregates in concrete, which mitigates the substantial volume deformation observed in cement paste [28]. The CTE of cement paste is between 8–28 × 10^−6^/°C, and its size depends on the water vapor ratio, aging, and moisture state. To calculate the CTE of concrete from the CTEs of the cement paste and aggregates, effective medium approaches such as the Rosen–Hashin bounds yield highly accurate results [29]. The process of crack formation in concrete structures is schematically illustrated in Figure 2 [1].

In practical applications, the CTE of aggregates is frequently disregarded, despite its significant role in determining the CTE of concrete. Consequently, supplementary cementitious materials (SCMs) are expected to exert a minimal influence on the CTE of concrete. Kou et al. [30] found that the CTE of ground granulated blast furnace slag (GGBS) cement concrete is 20% higher than that of Portland cement concrete. And CTE has a certain dependence on moisture [31,32]. During the initial stages of cement hydration, the CTE is very high, as the CTE of cement paste and water is similar. It gradually decreases to its lowest level as the cement solidifies [33].

Kamasamudram and Zahabizadeh et al. [34,35] found that CTE is almost constant from day one, but many studies [33,36,37,38,39,40,41] suggest that early hydration leads to an overall increase in concrete CTE. The CTE during concrete solidification is mainly controlled by its aggregates and will not undergo significant changes due to hydration. In fact, MacLeod et al. [42,43,44] discovered that cement hydration significantly influences the development of the CTE. They observed that when concrete is rewetted after several weeks of hardening, the CTE value decreases to the level measured at the time of cement solidification.

Based on the research conducted by Wyrzykowski et al. [32], the increase in CTE after solidification can be explained by establishing a relationship between the measured changes in internal relative humidity (ΔRH) and temperature (ΔT). Their findings suggest that the evolution of CTE is closely linked to moisture conditions within the cementitious matrix. The rise in initial CTE is anticipated to be more pronounced at lower w/b ratios, as cement concrete is more prone to self-drying in this situation. However, for cement pastes with a water-to-cement ratio (w/c) of up to 0.45, a significant increase in CTE was also measured, even though the increase in CTE in concrete due to self-desiccation was not as significant as in cement paste or mortar (due to the restraining effect of aggregates). Different scholars have found that the CTE growth rate of cement concrete ranges from 35% to 55% [45,46]. In practical scenarios, the CTE of concrete is commonly assumed to be a constant value in numerical computations. If not taken seriously, the increase rate of CTE is consistent with the above, and thermal cracking caused by temperature in concrete will have a huge impact. According to the research of many authors, the delayed thermal deformation in early concrete, potentially induced by changes in water state or water molecule transfer, holds significant importance for the study of concrete’s thermal shrinkage [47,48].

In order to explore the relationship between strength, temperature, and deformation, Saul et al. [49] established a non-stationary thermal equation to obtain a function of time and temperature intensity, in order to predict volume deformation [49], as shown in Equation (1):

(1) $$M=\sum_{0}^{t}\left(T-T_{0}\right)\Delta t$$

M: Maturity of age t;

T: Time interval Δ the average concrete temperature within t;

T_0_: The reference temperature, usually taken as −10 °C, representing the temperature at which no strength development is observed;

t: Over time;

Δt: Time interval.

Due to the inability to reproduce the experiment and insufficient accuracy of the data, a new maturity index equation is proposed according to the Arrhenius equation by Jensen et al. [50], as shown in Equation (2):

(2) $$t_{e}={\sum_{0}^{t}e}^{\frac{-Ea}{R}}\left(\frac{1}{T}-\frac{1}{Tr}\right)\Delta t$$

te: Equivalent age at reference temperature;

Ea: The apparent activation energy above 20 °C, which is 33.5 kJ/mol [51];

R: Universal gas constant;

T: Average absolute temperature of concrete during interval Δt;

Tr: Absolute reference temperature.

Equation (2) can also be utilized to assess other properties, such as self-shrinkage, creep, tensile strength, and Young modulus, as the maturity of concrete is contingent on factors such as the degree of hydration, temperature under load, and time. The maturity function equation can predict the autogenous shrinkage of concrete, but this prediction is flawed; even if the prediction of temperature change (semi-adiabatic) in the real environment is not completely accurate, this is because the self-shrinkage may be the viscoelastic deformation caused by the continuous pore pressure caused by self-drying, and the experimental conditions are too ideal.

The relative humidity of cement concrete with a low water–binder ratio (below 0.4) may decrease by 75–80% in a few days [52]. Cement hydration leads to the refinement of capillary pores, which become filled with hydration products. This process alters pore pressure and causes a reduction in internal relative humidity, a phenomenon known as self-desiccation. Self-drying causes a change in pore pressure that results in self-shrinkage. Tests on ordinary concrete have shown that autogenous shrinkage continues to develop as the strength increases. Once strength gain is complete, this shrinkage can generate tensile stresses within the concrete [53,54]. The degree of hydration of cement determines the activation energy of cement. In the hydration products, the activation energy of alite is 26–42 kJ/mol, and that of belite is 26–56 kJ/mol [55]. Typically, the activation energy of cement concrete cannot be directly computed, but an estimated value can be derived through experimental means. The addition of SCMs impacts the activation energy of cement concrete [56], and the addition of ground granulated blast furnace slag (GGBS) increases the apparent activation energy [57], whereas the addition of fly ash (FA) decreases the activation energy [58]. Many scholars have studied the autogenous shrinkage of concrete with SCMs and found that autogenous shrinkage and autogenous drying of concrete increase when silica fume (10–20%) replaces part of the cement [50]. The addition of FA will reduce the autogenous shrinkage; because of the low content of cement, the shrinkage rate of the whole concrete will increase in the later period [59]. In contrast to FA, the inclusion of GGBS enhances early-age self-shrinkage, promoting the formation of a finer pore structure and consequently increasing the self-desiccation of concrete [51].

The rate of autogenous shrinkage is contingent upon the extent of cement hydration, with elevated temperatures promoting hydration of the cement, because in cement concrete, an increase in temperature increases the internal RH, and the rate of hydration of the cement is faster in a high-RH environment. In concrete, RH in concrete decreases relatively when the temperature increases and then decreases to a low curing temperature, which will increase its self-shrinkage. Furthermore, when concrete cures at elevated temperatures, the pore structure tends to coarsen, which may ultimately reduce autogenous shrinkage. It is not yet possible to assess which role is dominant.

Autogenous shrinkage can induce self-induced stresses that exceed the tensile strength of concrete, a phenomenon governed by self-desiccation. Li et al. [60] developed a model for autogenous shrinkage that accounts for the creep and viscoelastic behavior of the solid skeleton and analyzed data from self-shrinkage and relative humidity results. This analysis suggests that long-term self-shrinkage may result from viscoelastic deformation caused by continuous pore pressure induced by self-drying. In order to evaluate the risk of thermal cracking in concrete, it is necessary to understand the changes in mechanical properties and volume, including the hydration process. The interaction between concrete and steel reinforcement has little correlation with understanding the development of temperature cracks. The initial physical characteristics of concrete progress during cement hydration. Concurrently, the exothermic reaction between water and cement, temperature fluctuations in the surrounding environment (including daily curing), and drying after demolding can impact the temperature elevation of the cement. It is necessary to understand the deformation caused by temperature changes for the application of concrete, especially at present; because SCMs exhibit lower heat release compared to cement, they have significant potential in concrete applications. Exploring the compatibility of PCE and SCMs (especially montmorillonite) and reducing the volume deformation of concrete are of great scientific significance.

#### MPCE Regulation Entry Point for Autogenous Shrinkage

The core inducement of autogenous shrinkage of concrete is the self-desiccation effect caused by cement hydration, which leads to a decrease in internal relative humidity, an increase in capillary tension of pore solution and the generation of autogenous shrinkage stress. In addition, the hydration rate of cement and the compatibility of SCMs and cement also affect the development degree of self-desiccation. MPCE realizes the targeted regulation of autogenous shrinkage from three core points: first, the polyurethane modified side chain can significantly reduce the surface tension of pore solution, directly weaken the capillary tension caused by self-desiccation, and reduce the autogenous shrinkage stress; second, MPCE has a high adsorption capacity on the surface of cement particles, which can appropriately regulate the hydration rate of cement, avoid the excessive consumption of free water caused by rapid hydration, and alleviate the self-desiccation effect; third, MPCE has good compatibility with SCMs such as fly ash and slag, which can cooperate with SCMs to refine the pore structure of concrete, reduce the number of capillary pores, and further inhibit the occurrence of autogenous shrinkage. The above regulation points are the key basis for MPCE to be superior to ordinary PCE in the control of concrete autogenous shrinkage.

### 2.2. Dry Shrinkage

**Figure 3:**
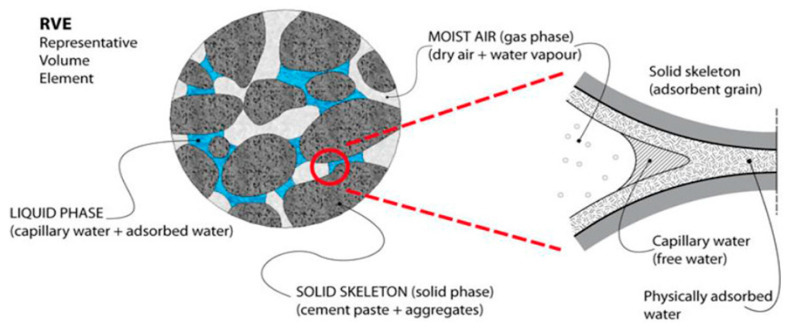
A schematic representation of the liquid/solid phase in concrete (adapted from [62]).

The definition of shrinkage is the volume deformation caused by changes in relative humidity inside and outside concrete. According to Nghia P. Tran [61], the change in capillary pressure within pore water was identified as another major mechanism of drying in porous media. The loss of moisture in the cement paste was found to disrupt the balance between water vapor and saturation pressure, leading to the formation of water–air menisci with varying radii of curvature in the capillary pores. These liquid–gas menisci (Figure 3), present in partially empty pores, were shown to induce hydraulic tensile stress in adjacent pore wall regions and generate isotropic compressive stress among the rigid solid skeleton, resulting in overall shrinkage.

Because the surface of concrete dries more rapidly than its interior, the resulting tensile stress can induce surface cracking. Such cracks facilitate the ingress of chlorides and sulfates, thereby compromising durability [63]. If regular maintenance is not carried out, the accelerated corrosion of cracks will quickly reduce the quality of concrete. Moreover, the magnitude of drying shrinkage varies among concrete mixtures with different w/c values. When w/c > 0.4, drying shrinkage dominates and accounts for a large proportion of the total shrinkage. In particular, when the w/c exceeds 0.42 (a critical threshold), the water content will exceed the complete hydration of cement [64]. As cement hydration progresses, this excess water will retain internal moisture, prevent self-drying, and gradually reduce the amplitude of self-shrinkage. Nevertheless, a high moisture content renders concrete susceptible to drying shrinkage resulting from physical moisture loss. When the w/c ratio exceeds 0.45, drying shrinkage is regarded as the primary factor driving the physical cracking of concrete during its service life [65].

The drying shrinkage of porous media (such as cementitious materials) is the result of internal moisture loss in porous structures, establishing a relative balance with the relative humidity (RH) of the external environment [66]. Hence, the magnitude of drying shrinkage in cement-based materials is primarily governed by the pore size distribution, the water-to-cement ratio, and the aggregate content, as these factors collectively determine the capillary pore structure, the availability of evaporable water, and the restraining effect of aggregates on volume change [67,68].

The volume deformation of concrete caused by shrinkage is mainly caused by four factors: the capillary tension effect, Gibbs–Bangham effect, separation pressure effect, and C-S-H interlayer water movement effect [69,70]. The contraction caused by these four dominant effects exists under different RH, and the Gibbs–Bangham effect mainly acts on RH < 40% [71,72]. In the relative humidity range of 40–50%, the Gibbs–Bangham effect and capillary tension act concurrently, with capillary tension gradually becoming dominant [73,74]. Within the 50–95% RH threshold, the disjoining pressure effect and interlayer water movement in C-S-H become operative [71]. Due to the fact that concrete is rarely exposed to environments with RH < 40%, and its overall RH is almost never lower than 40%, capillary tension effect is the main mechanism of its drying shrinkage.

**Figure 4:**
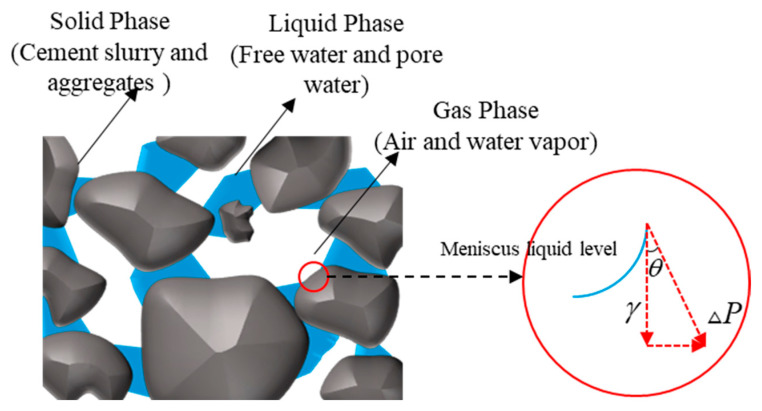
Schematic diagram of liquid and solid phases in concrete.

During the drying process of concrete, due to the destruction of the balance between steam and saturation pressure, the water in the capillary pores and gel pores of cement-based materials is constantly consumed, forming a meniscus with a continuously decreasing radius of curvature [69,74]. These curved interfaces generate hydrostatic tensile stress on neighboring walls and isotropic compressive stress between rigid solid frameworks within certain voids, causing volumetric shrinkage [75], as depicted in Figure 4. The magnitude of this additional pressure is directly related to the surface tension of the pore solution and can be explained by the Young–Laplace Equation (3):

(3) $$P=2\frac{\gamma }{r}$$

P: Additional pressure under the curved surface of capillary pores, MPa;

γ: Surface tension of capillary solution, N/m;

r: The curvature radius of the meniscus liquid surface for capillary pores, m.

Due to the hydrophilic nature of cementitious materials, water can completely wet the cement paste, and the contact angle is therefore typically assumed to be 0° [76,77]. The shrinkage resulting from capillary stress is entirely permanent, attributable to the collapse and closure of minuscule pores [78,79]. It is noteworthy that variations in capillary pressure are associated with internal relative humidity and pore size distribution, influenced by factors including but not limited to water content, supplementary cementitious materials, curing conditions, and degree of drying [75,80].

**Figure 5:**
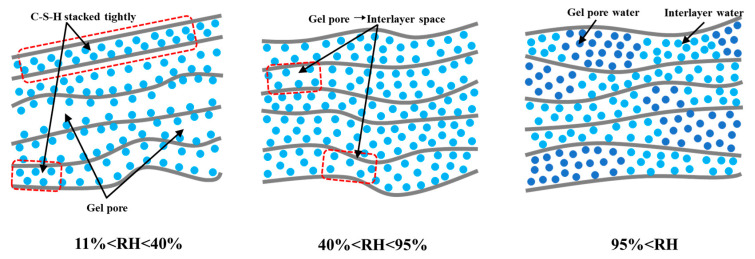
Changes in the arrangement of water between C-S-H layers during the drying process.

In addition, the interlayer water movement and separation pressure existing in C-S-H gel are the important factors that affect the later drying shrinkage of concrete. Within the range of RH > 50%, an increase in the thickness of the water film on the surface of overlapping C-S-H solids will generate repulsive forces, pushing away adjacent solid particles and balancing all interaction forces [81]. As pore humidity decreases, the desorption of water within the confined area causes a reduction in separation pressure. Thus, solid particles are brought closer together over a short distance due to van der Waals forces [75]. This phenomenon can also induce tensile stress in the nanopores and compressive stress on the rigid skeleton, resulting in long-term shrinkage following the drying process. At the same time, the partial release of water in the pores of C-S-H gel and the change in negative pressure in these pores make the distance between C-S-H layers change, resulting in the restriction of some gel pore water to interlayer water [82,83,84,85]. This interlayer water is only released at very low relative humidity because it strongly binds to the silicate-rich layer of solid C-S-H, as shown in Figure 5.

There are many ways to alleviate the shrinkage of concrete at present, such as using the characteristics of mineral admixtures such as slag, metakaolin, and fly ash to replace cement with appropriate proportions, which has a good shrinkage reduction effect [86,87,88,89]. Afroughsabet et al. [90] observed that substituting 30% of cement with slag led to a 56.4% decrease in drying shrinkage after 28 days of curing at a temperature of 20 °C. Li et al. [91] conducted a drying shrinkage test in laboratory conditions at 23 °C and discovered that incorporating 10% silica fume reduced the drying shrinkage strain by 34.9% after 30 days compared to ordinary concrete. Yang et al. used 10–20% metakaolin as a cement substitute and found that at a temperature of 23 °C, the drying shrinkage rate decreased by 13–18% at 23 °C and 50% relative humidity after 60 days [92]. In addition, adding shrinkage reducing agents and superplasticizer (such as polycarboxylate superplasticizer) can not only reduce the surface tension of the solution to prevent water loss in the solution but also block the hydration of cement particles, thereby controlling dry shrinkage [93,94], which will be explained in detail in the following section.

Polycarboxylate can significantly improve the flowability of concrete, making it more workable and pumpable. This characteristic is particularly important in concrete construction, as it ensures a uniform distribution of concrete during the pouring process, reduces the possibility of segregation and layering, and reduces labor intensity and construction costs [8]. Polycarboxylate has a significant improvement effect on the compressive and flexural strength of concrete. This improvement is attributed to the enhanced microstructure of concrete achieved by water-reducing agents, which decrease porosity, increase density, and subsequently enhance the strength and durability of the concrete. For some hydraulic structures that need to withstand heavy loads or harsh environments for a long time, such as dams or docks, the improvement of this performance is crucial. The use of polycarboxylate superplasticizer improves the adaptability of concrete to the external environment, including resistance to chloride ion corrosion, sulfate corrosion, and freeze–thaw cycles [95,96]. In addition, as polycarboxylate superplasticizer can effectively reduce the amount of cement used, this means that energy consumption and carbon emissions can be reduced in the production process of concrete, thereby improving the sustainability of concrete.

#### MPCE Regulation Entry Point for Drying Shrinkage

Drying shrinkage of concrete is dominated by the capillary tension effect (RH > 40%), and the migration of C-S-H gel interlayer water is the main factor leading to late drying shrinkage. The surface tension of the pore solution is the core physical quantity affecting capillary tension, and it is also the key target in MPCE regulation. For the main mechanism of drying shrinkage, MPCE exerts its shrinkage inhibition effect in two aspects: on the one hand, the modified polyurethane side chain can significantly reduce the surface tension of pore solution, directly reduce the capillary pressure according to the Young–Laplace equation, and inhibit the volume shrinkage caused by the capillary tension effect; on the other hand, MPCE molecules can adsorb on the surface of C-S-H gel, form a protective film on the gel surface, inhibit the excessive migration and desorption of interlayer water, and alleviate the late drying shrinkage caused by the change in separation pressure. In addition, MPCE can cooperate with mineral admixtures to refine the concrete pore structure, reduce the connectivity of capillary pores, slow down the loss rate of internal moisture, and further enhance the drying shrinkage inhibition effect. Compared with ordinary PCE and traditional shrinkage-reducing agents, MPCE integrates the functions of water reduction, surface tension reduction and gel protection and has a more comprehensive and effective regulation effect on drying shrinkage.

Beyond autogenous and drying shrinkage, exposure to sulfates and chlorides poses a serious threat to the structural parameters and strength of concrete with water-reducing admixtures. Chloride ions corrode steel reinforcement, leading to expansion and cracking, whereas sulfate ions generate expansive products (ettringite and gypsum) that induce internal stresses and strength deterioration. Properly designed polycarboxylate superplasticizers have been reported to mitigate such damage by refining pore structure and increasing compactness [97].

## 3. Molecular Design of PCE and Targeted Modification of MPCE for Volume Deformation Control

As one of the most important components in concrete construction today, polycarboxylate superplasticizer significantly improves the rheological and mechanical properties of concrete [98,99]. However, ordinary PCE has obvious shortcomings in the volume deformation control of concrete, struggling to meet the high requirements of hydraulic engineering for concrete shrinkage resistance. Therefore, based on the molecular design of ordinary PCE, targeted modification for the core mechanism of concrete volume deformation is the key to developing high-performance PCE. Polyurethane-modified polycarboxylate superplasticizer (MPCE) is designed by introducing polyurethane side chains to ordinary PCE, which optimizes the surface activity, adsorption capacity and compatibility with SCMs of PCE and realizes the targeted regulation of concrete volume deformation. Rheological studies have revealed that the incorporation of PCEs markedly reduces the shear stress of cementitious systems. For instance, Zhu et al. [100] found that the shear stress of a Portland–sulphoaluminate cement slurry decreases upon PCE addition, which is attributed to the combined electrostatic repulsion, lubrication, and steric hindrance effects of PCE molecules on cement particles. Similarly, Liu et al. [12] reported that the initial shear yield stress and apparent viscosity of cement paste are closely linked to the side-chain length and density of the PCE, with shorter side chains generally contributing to lower shear stress.

Using traditional PCE has certain limitations, including excessive set delay and low early strength development. In some concrete structures located in remote areas, it is essential for pre-mixed concrete to maintain high initial flowability to minimize flowability loss during long-distance transportation, thereby reducing the challenges associated with pumping and construction. To maintain concrete workability, the amount of PCE is often increased or mixed with other retarders. However, this can lead to early bleeding, which negatively impacts the concrete’s stability and performance at different temperatures. These limitations include undesirable interactions with other components in cementitious materials, such as clay or sulfate [13,14,15]; adverse effects on shrinkage cracking [101]; and the difficulty of meeting diverse performance requirements for concrete [23,102]. In terms of volume deformation control, the main limitations of ordinary PCE are that the surface activity is insufficient; the effect of reducing the surface tension of pore solution is limited; the adsorption capacity on cement particles is not high, and it is easily competitively adsorbed by SCMs, resulting in a reduced shrinkage inhibition effect; the molecular structure cannot effectively adsorb on the surface of C-S-H gel; and it is difficult to inhibit the migration of interlayer water and late drying shrinkage.

Polycarboxylate superplasticizers typically consist of a polyelectrolyte backbone and polyethylene glycol (PEG) side chains, a structure that can be tailored to specific applications. The PEG side chains and functional groups facilitate the dispersion of cement particles through electrostatic repulsion and steric hindrance [22,103]. Thus, by adjusting various components and polymerization processes, the molecular structure of PCEs can be tailored and modified to meet diverse structural requirements [104]. Over the years, many scholars have designed various new and efficient superplasticizer by changing their molecular structures and have studied their effects on cement-based materials [105,106,107,108]. PCEs can exert different steric hindrance effects through the modification of the length of their side chains. Additionally, the adsorption behavior of PCEs can be altered by incorporating various functional groups into the main chain or by adjusting the carboxylic acid density along the main chain [109,110,111].

Many scholars have studied the monomer molar ratio, side chain length, and modification of anionic anchoring groups in PCE [112,113,114,115,116,117]. Liu et al. [118] found that the use of new star-shaped PCEs can enhance the dispersion and flowability of cement paste, enhance its water reducing ability in mortar, and reduce saturation content. Zheng et al. [119] synthesized PCEs with branched, claw-like structures that offer strong steric hindrance and a “spherical effect,” along with enhanced surface activity and gas entrainment capabilities. Tan et al. and Zou et al. [120,121] discovered that PCEs with lower density, shorter side chains and higher molecular weight exhibit superior adsorption capacity on cement surfaces. Chen et al. [122] and Feng et al. [123] reported that changes in the bonding structure between the main and side chains of PCE affect the setting time and flowability retention of cement paste. Chen et al. [124] found that PCEs with longer side chains or shorter main chains function as stronger retarders and exhibit lower hydration levels.

In addition, there have been many studies on the performance of modified polycarboxylate superplasticizers with shrinkage-reducing functions (SRAs) in cement-based materials, which are designed through the modified molecular structure of PCE [125,126]. There have been works focusing on using various functional groups (including sulfonic and phosphate groups) to replace carboxylic acid groups on the main chain of PCE, in order to change the adsorption behavior and blocking ability of PCE [127,128]. Stecher [129] synthesized a series of innovative polyphosphate superplasticizers that significantly enhanced dispersion and demonstrated stronger resistance to sulfate and clay impurities. Akhlaghi [130] synthesized PCEs grafted with 2-acrylamido-2-methylpropanesulfonic acid (AMPS) onto the main chain of acrylic acid to produce low-density polyethylene glycol (PEG)-grafted chains. In ternary cementitious systems containing ordinary Portland cement (OPC) and calcined clay, the presence of high concentrations of sulfate ions prevents PCE from effectively intercalating into the layered structure of the clay while maintaining its molecular conformation. He et al. [131] demonstrated that enhancing the density of carboxylic acids and incorporating sulfonic acid groups into the main chain of PCEs can improve their adsorption capacity on the surface of cement particles, consequently leading to improved initial dispersion capabilities. Zuo et al. [26] prepared a new type of water-reducing agent and claimed that this new SRA has good water-reducing function but did not provide a detailed explanation. On the contrary, they observed that it could delay cement hydration and preserve internal relative humidity, consequently reducing early capillary pressure and self-shrinkage. Mao et al. [132] synthesized a novel polycarboxylate superplasticizer (SR-PCE) through the polymerization of selected monomers, including those with water-reducing properties, while reducing the overall number of monomer types used. Compared with traditional PCE, shrinkage-reducing polycarboxylate composite (SRPC) has weaker adsorption capacity and significantly delays cement hydration. It can mitigate self-shrinkage by reducing the surface tension of pore fluids, altering the pore size distribution to below 50 nm, and decreasing the rate of water evaporation [123,133]. However, previous studies have mainly focused on the shrinkage reduction ability of SRA and its alleviating mechanism on the shrinkage of cement-based materials.

**Figure 6:**
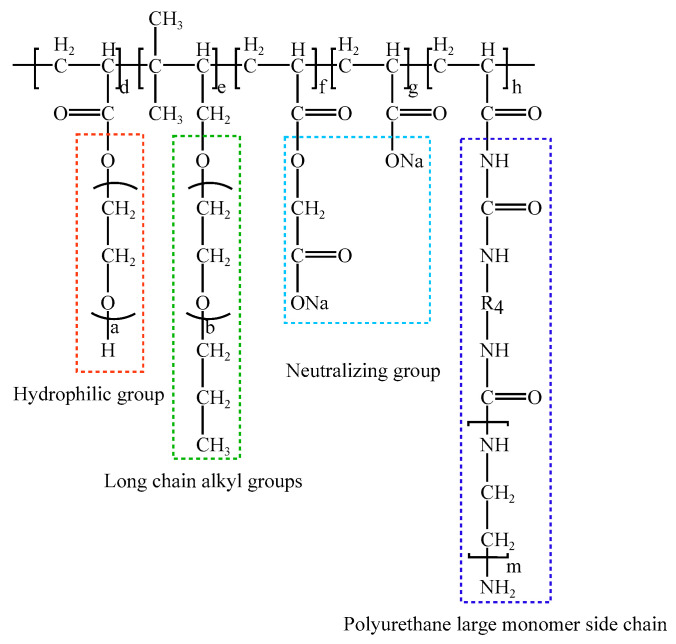
Molecular Structure of MPCE.

**Figure 7:**
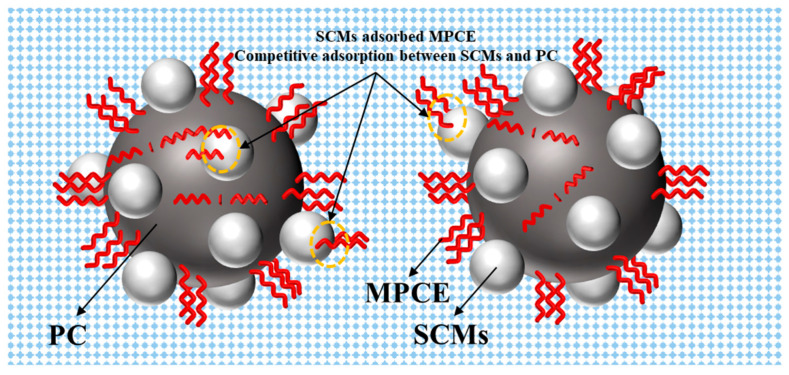
Competitive adsorption between MPCE and SCMs.

To address the limitations of ordinary PCE and SR-PCE in controlling concrete volume deformation, recent studies have developed a modified polycarboxylate superplasticizer (MPCE) by introducing polyurethane side chains into the PCE molecular structure through graft modification [132]. As illustrated in Figure 6, the molecular design of MPCE is specifically tailored to the core mechanisms of concrete volume deformation. According to the literature [132], the introduction of polyurethane side chains significantly enhances the surface activity of PCE molecules, which contributes to reducing the surface tension of the pore solution. In addition, the polyurethane side chains optimize the steric hindrance effect, improving adsorption onto cement particles and the C-S-H gel surface while mitigating competitive adsorption with SCMs, as illustrated in Figure 7. This modified molecular architecture retains the high water-reducing performance of conventional PCE while integrating workability improvement with effective volume deformation control.

The above research clarifies the molecular design of ordinary PCE, the limitations in volume deformation control, and the targeted modification strategy and molecular structure characteristics of MPCE. On this basis, the next chapter will present in-depth experimental research on the regulation effect of MPCE on the volume deformation of concrete, analyze the change law of MPCE on the surface tension of pore solution in cement paste containing SCMs, and reveal the intrinsic mechanism behind MPCE inhibiting autogenous shrinkage and drying shrinkage.

## 4. Control of Volume Deformation of Concrete by PCE

An effective solution for suppressing shrinkage cracks is to incorporate shrinkage-reducing admixtures (SRAs), which control shrinkage by reducing the surface tension of the pore solution [23,24,25]. However, Bentz et al. [134,135] observed that the addition of SRA delayed cement hydration and also inhibited the development of cementitious material strength. Scholars have also found that the addition of SRA reduces the air content in concrete, thereby reducing its frost resistance [136,137,138,139,140,141]. Furthermore, the poor compatibility between SRA and PCE can diminish the effectiveness of the SRA. Hence, it is essential to develop a more suitable approach to overcome these challenges [142,143,144,145,146,147,148,149,150].

**Figure 8:**
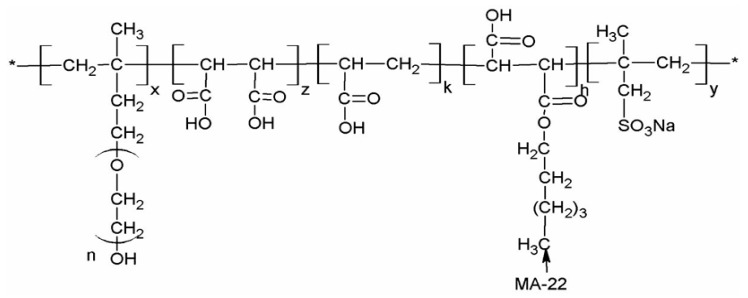
Chemical structure of SRPC. (* denotes the linking site to the polymer backbone).

Many scholars have conducted grafting and modification designs in PCE and SRA to enhance their shrinkage reduction ability. Mao et al. [132] designed a highly efficient polycarboxylate superplasticizer (SR-PCE) by grafting reducing groups onto the main chain alongside carboxyl groups. The chemical structure of SR-PCE is shown in Figure 8.

**Figure 9:**
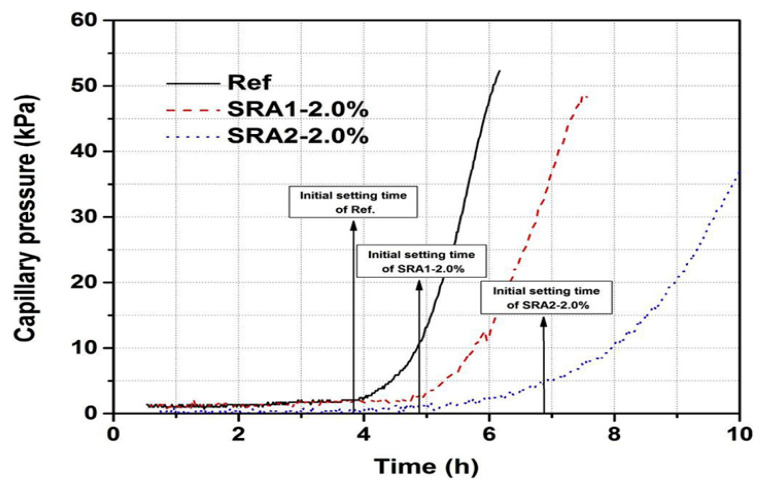
Development of very-early-age capillary pressure of cement pastes as a function of time (adapted from [151]).

In recent years, numerous studies have investigated the performance of SR-PCE in cementitious materials, following its synthesis through copolymerization of unsaturated acids and reducing functional monomers, which effectively resolves the compatibility issues between PCE and SRAs. Zuo et al. [26] found that polyether-type SRAs significantly delay cement hydration, leading to a sustained high internal RH within the cement paste. Data illustrating the development of capillary pressure at a very early age in cement pastes, both with and without SRAs, are presented in Figure 9.

Consequently, this effectively reduces both capillary stress and self-shrinkage. However, the water-reducing performance of polyether-type SRAs is not satisfactory, as they primarily function as shrinkage-reducing admixtures rather than a highly efficient polycarboxylate superplasticizer. Yang et al. [152] examined the impact of SR-PCE on reducing cracking mechanisms using a temperature stress testing machine. To achieve the desired performance, this shrinkage-reducing PCE needs to be combined with a high-efficiency polycarboxylate superplasticizer, which means its water-reducing performance is poor. Ran et al. [125] designed a multifunctional and efficient water-reducing agent JM-PCA (a type of shrinkage-reducing polycarboxylate superplasticizer) with a shrinkage reduction function. However, there is still room for improvement in its shrinkage reduction capabilities. Mao et al. [153] stated that the new shrinkage-reducing polycarboxylate, SR-PCE, delays cement hydration. However, they noted that SR-PCE’s adsorption capacity is not as high as that of traditional PCE, and the shrinkage reduction ability is much better than traditional PCE. In addition, the reduction ability of SR-PCE may be attributed to reducing surface tension and adjusting pore size distribution.

Similar to SRAs, the capillary tension theory is commonly employed to elucidate the mechanism behind the contraction reduction in SR-PCE, offering significant insights into the surface tension of pore solutions [132,154]. However, another crucial property of the pore solution that influences volume deformation is the contact angle between the pore wall and the pore solution. Modifying the contact angle by adding polymers can change the capillary stress within porous media, thus impacting shrinkage deformation. Bentz et al. [134] reported that the introduction of SRA reduced the contact angle from 28° to 7°. Wang et al. [155] discovered that an increased contact angle contributes to the shrinkage reduction effect of SRAs. The variability in contact angle changes after polymer incorporation can largely be attributed to differences in polymer structure and properties. In the following section, we summarize the key experimental findings reported in the literature to elucidate the underlying mechanisms.

**Figure 10:**
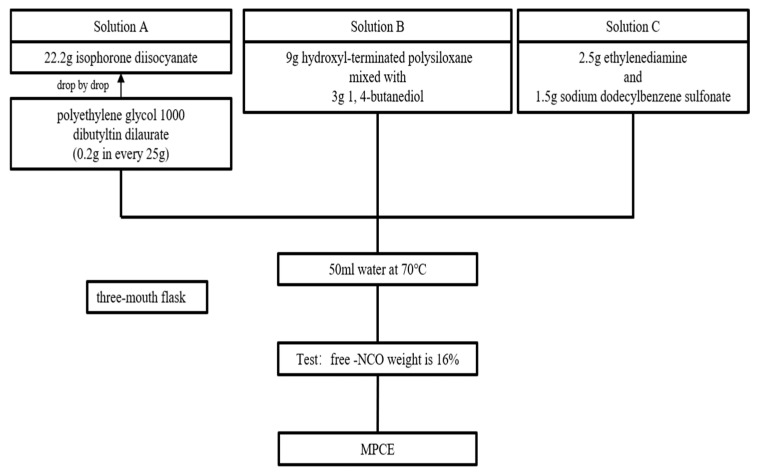
Synthesis of MPCE.

In summary, the shrinkage or water-reducing properties of the above-mentioned chemical admixtures are not sufficient to be used solely for cement-based materials. The development of SR-PCEs with superior shrinkage and water reduction properties remains a significant research focus. The introduction and application of MPCE can address specific gaps in this field. The synthesis steps and methods are illustrated in Figure 10. It should be emphasized that the synthetic procedure illustrated in Figure 10 is based on and extends the work of Xiang et al. [156], who first reported the preparation of MPCE; this figure does not propose any new testing standard.

### 4.1. Materials and Methods

Xiang et al. [156] conducted experiments to verify the effect of MPCE on volume deformation control in concrete. In their study, P·O 42.5 ordinary Portland cement was used as the binder. Three types of supplementary cementitious materials (SCMs)—fly ash (FA), ground granulated blast furnace slag (GGBS), and metakaolin (MK)—were added at replacement rates of 10%, 20%, 30%, and 40% by mass of cement. A commercial ordinary polycarboxylate superplasticizer (OPCE) and two unmodified polycarboxylate mother liquors (C-PCE1, C-PCE2) were used as controls. MPCE was synthesized according to the method reported in [155].

The water-to-cement ratio (w/c) of all cement pastes was fixed at 0.35. The dosage of each PCE type was 0.5% by mass of cement. After thorough mixing, the fresh paste was centrifuged to obtain the supernatant. The surface tension of the supernatant was measured using a fully automatic surface tension meter (A-60, Kino Industry Co., Ltd., Washington, DC, USA). The adsorption amount of PCE onto cement particles was determined by the total organic carbon (TOC) method, measuring the concentration difference before and after centrifugation.

### 4.2. Adsorption Capacity and Surface Tension

**Figure 11:**
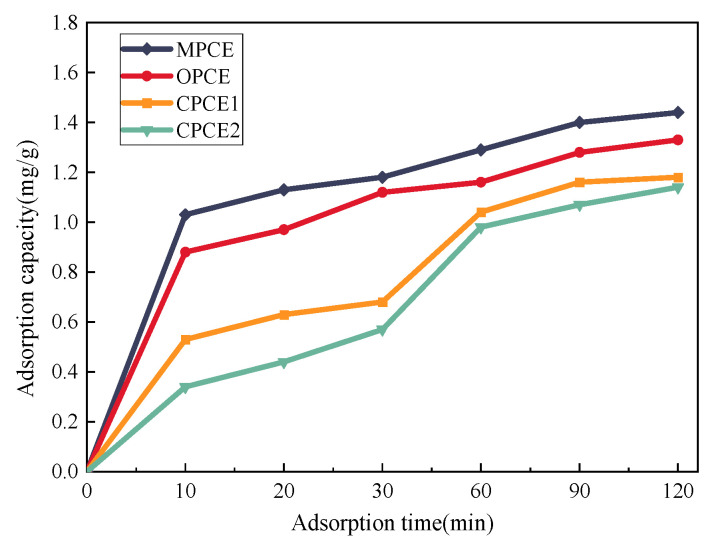
Adsorption capacity of PCE.

According to the results reported by Xiang et al. [156], the adsorption capacity of MPCE on cement particles was compared with that of OPCE, C-PCE1, and C-PCE2. The results, presented in Figure 11, show the evolution of adsorbed amount over time.

The significantly higher adsorption capacity of MPCE compared to the other three PCEs is mainly attributed to the structural advantages of its polyurethane side chains. The polyurethane side chains provide stronger steric hindrance, effectively preventing PCE molecules from lying flat on the cement particle surface, thus preserving more effective adsorption sites. In the presence of metakaolin, the decrease in MPCE adsorption is smaller than that of other PCEs, because the polar groups of the polyurethane side chains have weaker interactions with the layered structure of metakaolin, reducing intercalation consumption.

Within 10 min, the adsorption capacities of all four types of PCEs increased sharply, accounting for about two thirds of the saturated adsorption capacity. Among them, the adsorption capacity of MPCE was 117.1%, 194.3%, and 302.9% of OPCE, CPCE1, and CPCE2. After 60 min, the adsorption capacity of the four types of PCEs gradually stabilized. In addition, it was found that the addition of auxiliary cementitious materials would affect the adsorption capacity of PCE in the same dosage of cement, due to the fact that MPCE can reduce capillary stress by reducing the surface tension of pore solutions, thereby reducing concrete shrinkage [60].

Therefore, fly ash, slag, and metakaolin were each added to cement pastes containing different dosages of MPCE, with cement substitution rates of 10%, 20%, 30%, and 40% for each type of SCM. After thorough stirring, centrifugation was carried out to obtain the upper clear liquid, and the surface tension changes were measured using a fully automatic surface tension meter A-60 (Washington, USA), as shown in Figure 11.

Compared with conventional shrinkage-reducing admixtures (SRAs) reported in the literature, MPCE exhibits superior performance in reducing surface tension. Bentz et al. [134] reported that SRAs could reduce the surface tension of the pore solution by approximately 20–25%, but they also delayed cement hydration and reduced early strength. In contrast, MPCE achieved a 10% reduction in surface tension without noticeable retardation, owing to the optimized design of polyurethane side chains. Compared with JM-PCA reported by Ran et al. [125], MPCE shows better compatibility with SCMs; even in the presence of metakaolin, MPCE maintains a relatively high adsorption capacity (Figure 11). This indicates that the polyurethane side chains effectively inhibit competitive adsorption with montmorillonite-type minerals.

Unlike conventional shrinkage-reducing admixtures (SRAs) reported in the literature, MPCE does not cause significant retardation while reducing surface tension. This is attributed to the unique structure of the polyurethane side chains: their flexible segments allow rapid adsorption onto cement particle surfaces without strongly chelating calcium ions or encapsulating cement particles, thereby avoiding significant delays in the cement hydration process.

**Figure 12:**
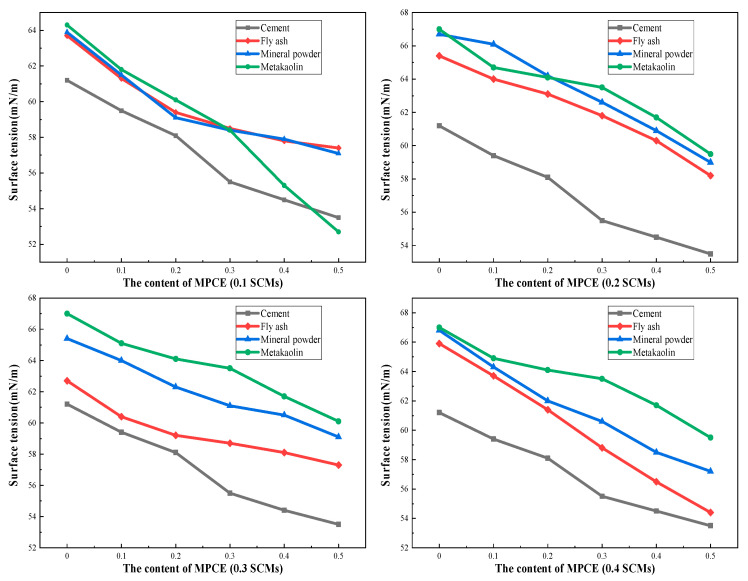
Surface tension of MPCE in cement paste containing SCMs.

As shown in Figure 12, with the increase in MPCE content, the liquid phase surface tension of freshly mixed cement paste continuously decreases, with a minimum decrease of 10% [132]. This indicates that with the increase in MPCE content, more and more polycarboxylate molecules are not effectively adsorbed by cement particles and thus remain in the liquid phase, resulting in a decreasing trend in the measured surface tension of the liquid phase. With the addition of auxiliary cementitious materials, the liquid phase surface tension of fresh concrete also shows a decreasing trend. As the content of the admixture increases, the decrease in surface tension becomes greater. This is because the particle size of the mixed mineral powder and fly ash is smaller than that of Portland cement. These materials consist primarily of aluminosilicate glass microspheres with a sponge-like texture, resembling glass beads in appearance. They are characterized by a small average particle size, low internal specific surface area, dense microstructure, smooth surface texture, excellent flowability, and minimal free water adsorption. They play a role as “ball bearings” in concrete mixtures [157,158,159,160,161,162,163,164,165], which can better fill the gaps of silicate cement hydration products, greatly reduce internal porosity, and refine pore size [166,167,168,169,170,171,172,173]. Among the four types of SCMs, the surface tension of the upper clear liquid after adding metakaolin is the highest, indicating that the amount of MPCE adsorbed on the surface of cement particles is less, and more remains in the solution. This is because metakaolin has the strongest adsorption capacity for MPCE. As reported by Xiang et al. [156], its unique layered structure allows the polyether side chains of polycarboxylates to be effectively intercalated into the layers, competing with cement particles for adsorption. Consequently, MPCE cannot be effectively adsorbed onto the surface of cement particles when metakaolin is present. In addition, after adding auxiliary cementitious materials, the solubility of the admixture is lower than that of the silicate cement particles, resulting in a simultaneous decrease in the concentration of Ca^2+^, Na^+^, and OH^−^ ions in the pore solution. In the early hydration process of Portland cement, the Si-O bond will break in an alkaline environment and then combine with hydrated and dissociated ions to form C-S-H gel, which leads to a reduction in counter ion concentration on the sliding surface of Portland cement particles [174,175,176,177,178,179,180,181,182]. Therefore, MPCE reduces the surface tension of the pore solution in concrete rich in auxiliary cementitious materials, thereby regulating its volume deformation.

## 5. Conclusions

This review systematically investigates the mechanisms and application performance of polyurethane-modified polycarboxylate superplasticizer (MPCE) for volume deformation control in hydraulic concrete. The main conclusions are as follows:

(1) Compared with ordinary PCE, MPCE significantly reduces the surface tension of the pore solution in cement paste (by up to 10%) through the introduction of polyurethane side chains, thereby directly weakening capillary tension and effectively inhibiting both autogenous and drying shrinkage in concrete.
(2) MPCE exhibits superior adsorption performance: at the same dosage, its adsorption amount on cement particles is 117.1%, 194.3%, and 302.9% that of ordinary PCE, C-PCE1, and C-PCE2, respectively. Furthermore, MPCE shows good compatibility with supplementary cementitious materials (fly ash, slag, metakaolin), enabling synergistic refinement of pore structure and further enhancing shrinkage reduction.
(3) The dual mechanism of MPCE is elucidated: on one hand, it reduces capillary pressure by lowering the surface tension of the pore solution; on the other hand, it adsorbs onto the C-S-H gel surface, inhibiting interlayer water migration and mitigating late-age drying shrinkage.
(4) For the design of hydraulic concrete structures incorporating MPCE, it is recommended that engineers account for the reduced autogenous and drying shrinkage when determining crack control spacing and reinforcement ratios. Additionally, the lower surface tension of the pore solution may affect the bond strength between concrete and reinforcement, which should be verified through structural-scale tests. Given the improved compatibility with supplementary cementitious materials, MPCE-containing concrete is particularly suitable for mass concrete elements where thermal and shrinkage cracking are critical concerns.

Future research should further explore the effects of polyurethane side chains with different lengths, densities, and functional group modifications on MPCE performance and conduct long-term durability studies to promote the engineering application of MPCE in high-performance hydraulic concrete.

## Data Availability

No new data were created or analyzed in this study. Data sharing is not applicable to this article.
